# Characterization of the five *Amanita* complete mitochondrial genome and the phylogenetic relationship with other *Amanita* fungi

**DOI:** 10.1186/s12864-025-12103-w

**Published:** 2025-09-29

**Authors:** Xianyi Wang, Guangyin Xu, Jiawei Tao, Guoyu Wang, Zhongyao Guo, Huangxue Luo, Guihong Li, Hongmei Liu, Chunying Deng, Yuanming Wu

**Affiliations:** 1 Engineering Research Center of Medical Biotechnology, School of Biology and Engineering, Guizhou Key Laboratory of Microbio and Infectious Disease Prevention & Control, Department of Parasitology, School of Basic Medical Sciences, Guizhou Medical University, Guiyang, 561113 China; 2 Key Laboratory of Biology and Medical Engineering, Immune Cells and Antibody Engineering Research Center of Guizhou Province, School of Biology and Engineering, Guizhou Medical University, Guiyang, China; 3 Department of Scientific Research Management, Guizhou Academy of Sciences, Guiyang, 550001 China

**Keywords:** *Amanita*, Mitogenome, Mitogenome, Poisonous mushroom, Molecular phylogeny, Evolution, Diversity, Identification

## Abstract

**Background:**

*Amanita* is a large genus of mushrooms with a rich biodiversity. It includes the world’s most poisonous mushroom, which is responsible for 90% of mushroom-related fatalities. However, it is difficult to distinguish lethal and edible *Amanita* species because of their morphological similarities.

**Results:**

To identify molecular data and explore the phylogenetic relationships within *Amanita*, we sequenced and analyzed the mitochondrial genome (mitogenome) of five *Amanita* species and compared them with nine previously published *Amanita* mitogenomes. We analyzed the genomic structures, tandem repeats and gene rearrangements, introns, adenine–thymine (AT) skew, guanine–cytosine (GC) skew, and transfer RNAs (tRNAs) of the five *Amanita* species. Phylogenetic trees were constructed among 55 species from Agaricomycetes, including 14 species of *Amanita*, 39 species of Agaricales, and two outgroup species of Auriculariales, based on two different datasets (15 protein-encoding genes [PCGs] of amino acids and 15 PCGs of codons). Phylogenetic analysis revealed that the 14 *Amanita* species had consistent phylogenetic positions with the current phylogeny. *Amanita* was divided into three subgenera (subgenus *Amanita*, subgenus *Amanitina*, subgenus *Lepidella*). Moreover, the phylogenetic analysis confirmed that ectomycorrhizal and asymbiotic species clustered into different subgenera.

**Conclusions:**

We described the features of *Amanita* mitogenome, including the genomic structures, tandem repeats and the rearrangement of the PCGs related to classification status, which showed a different arrangement of genes and clear rearrangement with the introns between the ectomycorrhizal and asymbiotic. Finally, we proposed that the subordinate taxon of *Amanita* requires further revision. This study provides insights into the evolution and structural characteristics of the mitogenomes of macrofungal organisms.

**Supplementary Information:**

The online version contains supplementary material available at 10.1186/s12864-025-12103-w.

## Background

*Amanita* belongs to Amanitaceae, Agaricales, and Basidiomycetes. The genus is rich in species of Amanitaceae has been described in the literature as well as online (http://www.amanitaceae.org/), with approximately 1,200 species worldwide, of which more than 650 are considered good species [1, 2]. *Amanita* is widely distributed worldwide and exhibits a variety of characteristics. These species are primarily symbiotic with Fagaceae and Pinaceae occurring in the mixed forest of tropical and subtropical regions [3, 4]. Moreover, *Amanita* has a close relationship with humans as edible *Amanita* has a high nutrient content and medicinal value; however, some species are poisonous and may be fatal. In fact, 90% fatality of mushroom intoxication is caused by the consumption of poisonous *Amanita* [1, 5–9].

Based on morphological studies and molecular phylogenetic analyses, several classifications for *Amanita* have been proposed; however, they show no unified results. Gay [10] and Roze [11] separated the species of the sterile annulus from the genus of *Amanita* into a new genus. Gilbert [12] divided *Amanita* into three subgenera: *Amanita*, *Amanitopsis*, and *Limacella*. Gilbert and Kühner et al. [13] divided *Amanita* into two categories based on whether it has spore starch and veins on the edge of the pileus. Vesel [14] divided *Amanita* into three subgenera: *Euamanita*, *Amanitopsis*, and *Lepidella*. Corner and Bas [15] and Bas [16] separated *Amanita* into six groups of two subgenera. Yang [17] and Weiss et al. [18] divided *Amanita* into seven section*s*. Currently, *Amanita* is divided into three subgenera and 11 sections, including the subgenus *Amanita* (sect. *Amanita*, sect. *Amarrendiae*, sect. *Caesareae*, and sect. *Vaginatae*), subgenus *Amanitina* (sect. *Amidella*, sect. *Arenariae*, sect. *Phalloideae*, sect. *Roanokenses*, sect. *Strobiliformes*, and sect. *Validae*), and subgenus *Lepidella* (sect. *Lepidella*) [1]. *Amanita* species contain multiple variant forms, and determining the taxonomic status of these subunits remains challenging [19]. The genus *Amanita* exhibits rich species diversity, with widespread distribution and numerous new species, varieties, cryptic species, difficult species, and undiscovered species [20].

The mitochondrial genome (mitogenome) has many important functional genes. It also exhibits fast evolution, strict maternal inheritance, and a small genome. As a nuclear genetic material, it has many characteristics that are different from nuclear genes and has been widely applied in phylogenetic research. Li et al. [21] compared and analyzed three species of *Cantharellus* and found that the mitogenome in different regions could be used to construct a phylogenetic tree Based on the analysis of 15 protein-coding genes. *C*ytochrome b (cob), cytochrome oxidase subunit 1 (*cox1*), and *16 S* ribosomal RNA (*rnl*) were used as single gene markers to analyze the phylogenetic relationship between species, and the mitogenome was found to be helpful in studying population genetics and fungal evolution. Based on the mitogenome, they [22] established the phylogenetic relationship among 66 Basidiomycetes and revealed mitochondrial function in the evolution and ecological adaptation of *Amanita*. Molecular phylogenetic studies based on gene fragments can provide insights into the evolutionary history of *Amanita* [23].

Complete mitogenome sequencing and a faster evolution rate are more suitable for the determination of their taxonomic status. Introns with a fast evolution rate and low similarity are present in the mitogenome of *Amanita*; these are more suitable for tracing the origin of *Amanita* [21].

In this study, we sequenced and annotated the mitogenome of 14 *Amanita* species (5 species from this study and nine from NCBI). We analyzed genomic characteristics, including genome size, repetitive sequences, gene sequences, and amino acid use, and constructed a phylogenetic tree to verify the classification status. The results of this study further deepen our understanding of the genetic and evolutionary relationships of *Amanita*.

## Materials and methods

### Sample collection, species morphological identification, and DNA extraction

The information of five *Amanita* specimens (*A*. *eijii*, *A*. *pallidorosea*, *A*. *oberwinklerana*, *A*. *sinocitrina*, and *A*. *rimosa*, collection details in Table 1) was initially recognized by morphological studies based on color, pileus, annulus, stipe, volval remnant, and microscopic spore characteristics. Genomic DNA was extracted using a fungi genomic DNA extraction kit (Beijing Solarbio Science & Technology Co., Ltd.) based on the manufacturer’s instructions. The extracts were stored in a − 20 °C refrigerator. The purity and quality of genomic DNA were gauged using a Thermo Scientific ultra-trace UV–visible photometer.

**Table 1:** Information of the samples used in this study

Species	Collector	Locus	Time
*Amanita eijii*	Chunying Deng	Qiannan, Guizhou Province, China	August 2022
*Amanita oberwinklerana*	Jiawei Tao	Tongren, Guizhou Province, China	August 2020
*Amanita pallidorosea*	Xianyi Wang	Tongren, Guizhou Province, China	August 2020
*Amanita rimosa*	Zhongyao Guo	Tongren, Guizhou Province, China	July 2020
*Amanita sinocitrina*	Chunying Deng	Tongren, Guizhou Province, China	July 2020

### Sequencing and species molecular identification

Genomic DNA from the abovementioned five *Amanita* species was analyzed via next-generation sequencing (NGS) using Illumina HiSeq 4000, Berry Genomics, Beijing, China; raw data were obtained from 150-bp paired-end reads [24]. The specimens were identified Based on molecular fragment identification. We assembled the internal transcribed spacers 1 (*ITS1*) for the five *Amanita* species using homologous sequences (NR_189951.1) [25], BLAST search of the NCBI database, and the pairwise alignment function of Geneious R9 [26].

### Genome assembly and annotation

Raw NGS reads were imported into Geneious R9 and paired files were set to the relative orientation with the forward/reverse primers pointing inward, with an insert of 500 bp. The reads were trimmed and normalized using BBDuk Trimmer (Paired Read Merger Version 38.84) (Supplementary Material Table S1). Mitogenome assemblies were acquired using the “map to reference” assembly and “De novo assembly” in Geneious R9 and compared with the reference sequences of *A*. *basii* (NC 045195) and *A*. *pseudoporphyria* (NC 045194) [22, 26] as reference sequences with custom sensitivity. The reference assembly was set to “none” (fast/read mapping), and the mitogenome represented by at least five iterations (map reads to the consensus from the previous iteration) assembled the fastq paired-end sequence datasets. Paired reads had a maximum per read of 10% and a maximum gap size of 15. The index word length was set to 13, maximum mismatches per read were set to 1%, minimum overlap identity was set to 99%, and maximum ambiguity was set to 2. Accurately selected map reads with errors in the repeat regions were, then identified through BLAST searches of the NCBI database. We predicted the basic positions of the tRNAs using the MITOS Web Server (http://mitos.bioinf.uni-leipzig.de/index.py) [27, 28]. Protein-coding genes and the large ribosomal subunit (*rnl*) and small ribosomal subunit (*rns*) were annotated using homologous sequences and the open reading frame tool finder (ORF) of Geneious R9 [26]. Finally, all data have been submitted to NCBI, where their accession numbers are as follows: *A. eijii* (PP409005), *A. oberwinklerana* (PP409003), *A. pallidorosea* (PP978973), *A. rimosa* (PP978972), and *A*. *sinocitrina* (PP409004).

### Sequence analysis

We used Repeats Finder to identify the tandem repeats of five *Amanita* species [29]. Genetic collinearity was determined using the Mauve of Geneious R9 [26, 30]. The secondary structure of tRNA was predicted using the MITOS Web Server and Adobe Illustrator 2020 for image production [28]. The mitogenome maps were constructed using CGView tools of Proksee (https://proksee.ca/*)* and Adobe Illustrator 2020 [27, 31]. The Base composition, amino acid composition, and relative synonymous codon usage were calculated using MEGA 7 [32]. The base usage preference was calculated according to the following formulas: AT skew = (A − T)/(A + T) and GC skew = (G − C)/(G + C) [28].

### Phylogenetic analysis

To examine the phylogenetic relationship among different sections of *Amanita* and its phylogenetic position in Agaricales, we reconstructed the phylogenetic tree of 14 *Amanita* species (*Stropharia rugosoannulata* and *Coprinellus micaceus* as the outgroup) and combined 41 species downloaded from NCBI. In total, 53 species of Agaricales and 2 outgroup species of Auriculariales were included (Supplementary Material Table S2). Two datasets were obtained from all of the amino acids and codons of the 15 PCGs (*atp6*: *ATPase subunit 6*, *atp8*: *ATPase subunit 8*, *atp9*: *ATPase subunit 9*, *cob: apocytochrome b*, *cox1–3*: *Cytochrome oxidase subunit 1–3*, *nad 1 − 6*: *NADH dehydrogenase subunit 1* − *6*, *nad4L*: *NADH dehydrogenase subunit 4 L*, and *rps3*: *ribosomal protein S3*), which were extracted separately from Geneious R9 [26] and aligned using MEGA 7 [32]. Base composition Heterogeneity in the 15 PCG dataset was analyzed using Aligroove software, which preliminarily tested the reliability of these two datasets [33]. Maximum likelihood (ML) analysis was performed using IQ-TREE (http://iqtree.cibiv.univie.ac.at/) with 1,000 bootstrap replicates to estimate node support using the ultrafast bootstrap approach [34]. Bayesian inference (BI) analysis was performed using MrBayes 3.2.6 [35] with a sampling of 1,000 generations to imitate four independent runs for 1 million generations. We reduced the average standard deviation of the split frequency to < 0.01, abandoned the initial 25% burn-in samples, generated a consensus tree, and calculated the posterior probabilities. The phylogenetic tree was visualized and decorated using FigTree v1.4.3 and Adobe Illustrator 2020 [36].

## Results

### Genomic structure and organization

**Fig. 1:**
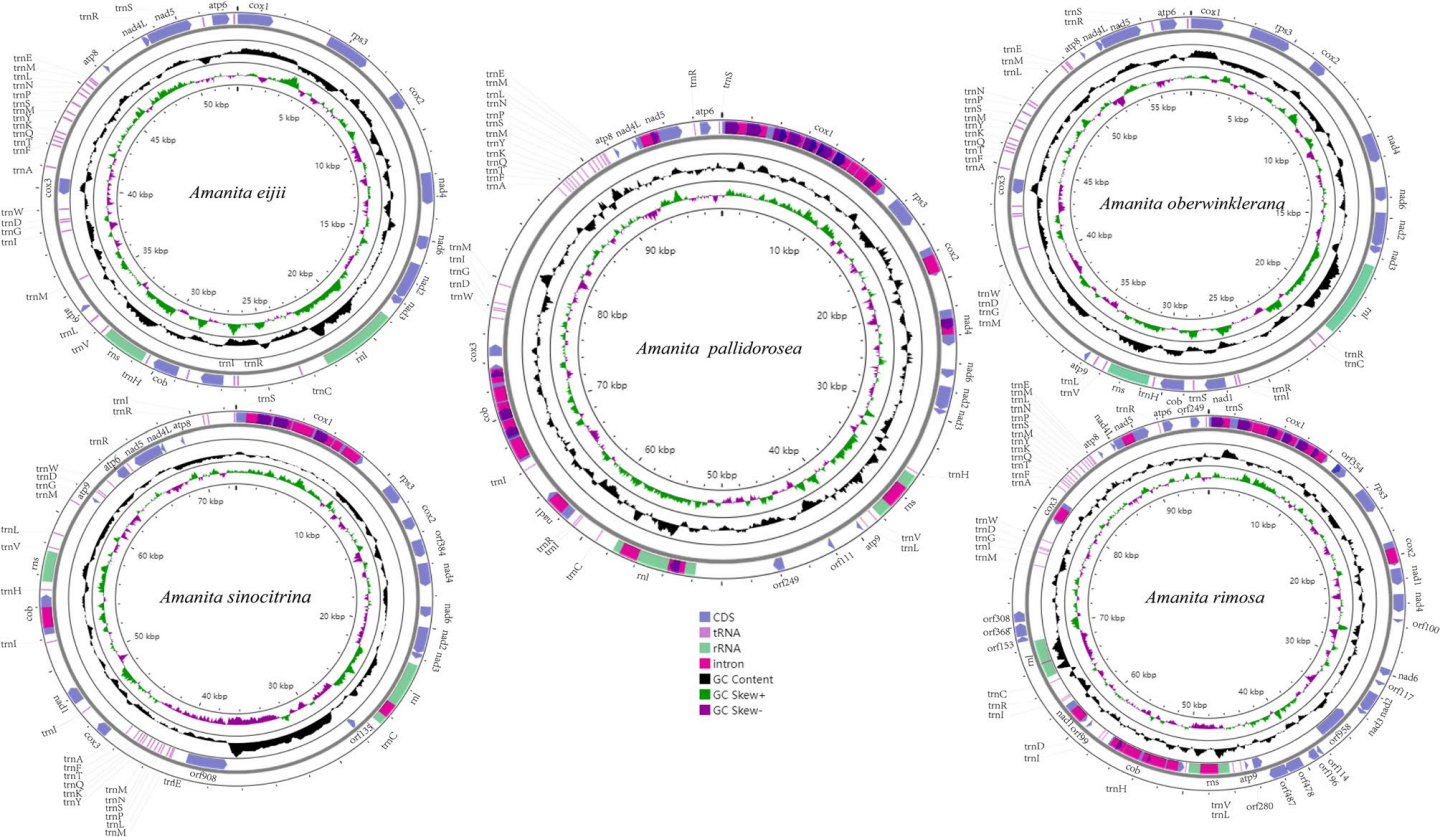
Circular maps of the mitogenomes of five *Amanita* species. Different colored blocks indicate the different genes. The colored blocks on the outside ring indicate the genes on the direct strand, and the gene-colored blocks on the inside ring indicate the genes on the reverse strand. The lines outside of the ring correspond to the names of the genes

**Fig. 2:**
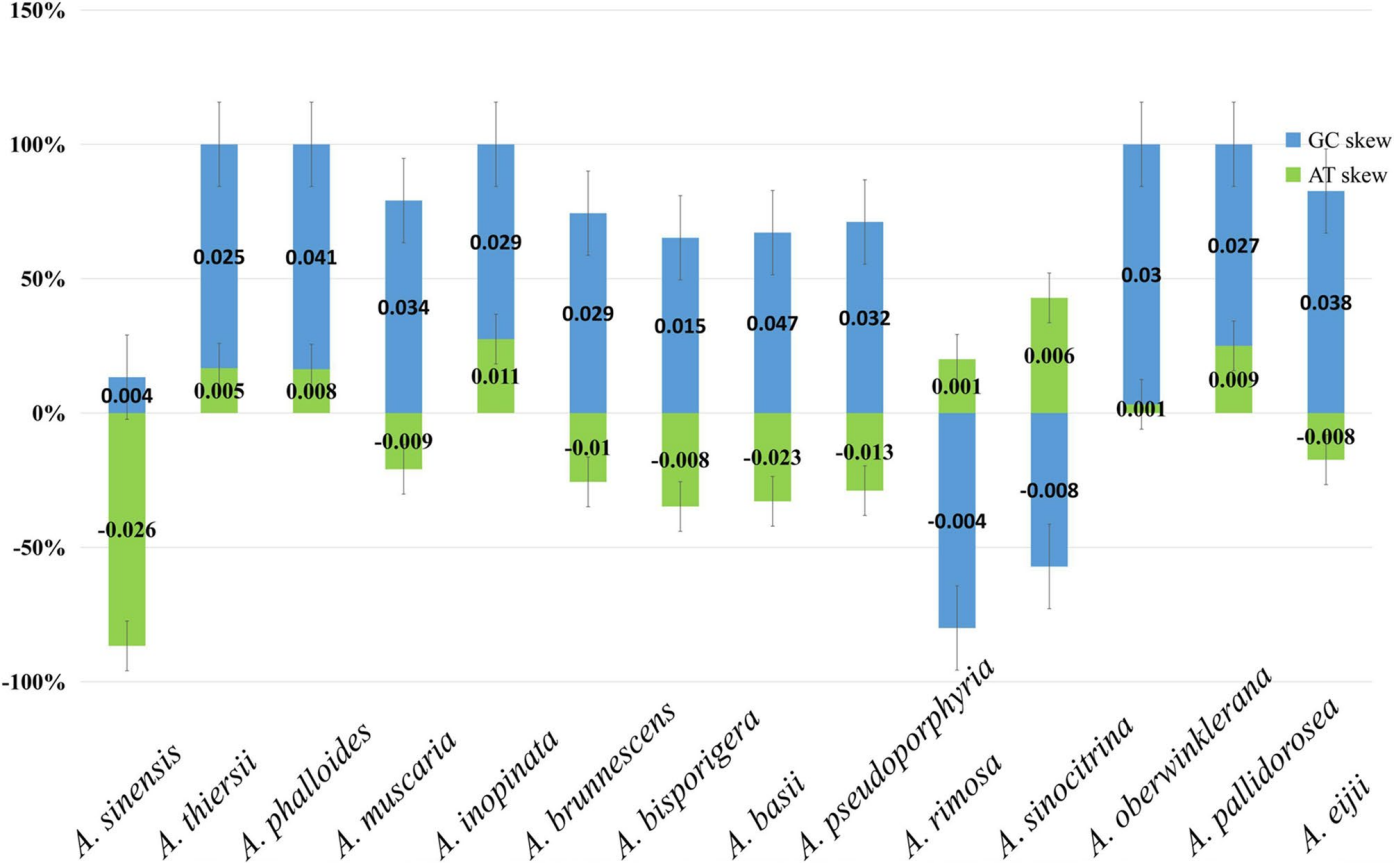
AT and GC skews of the 14 *Amanita* species

**Fig. 3:**
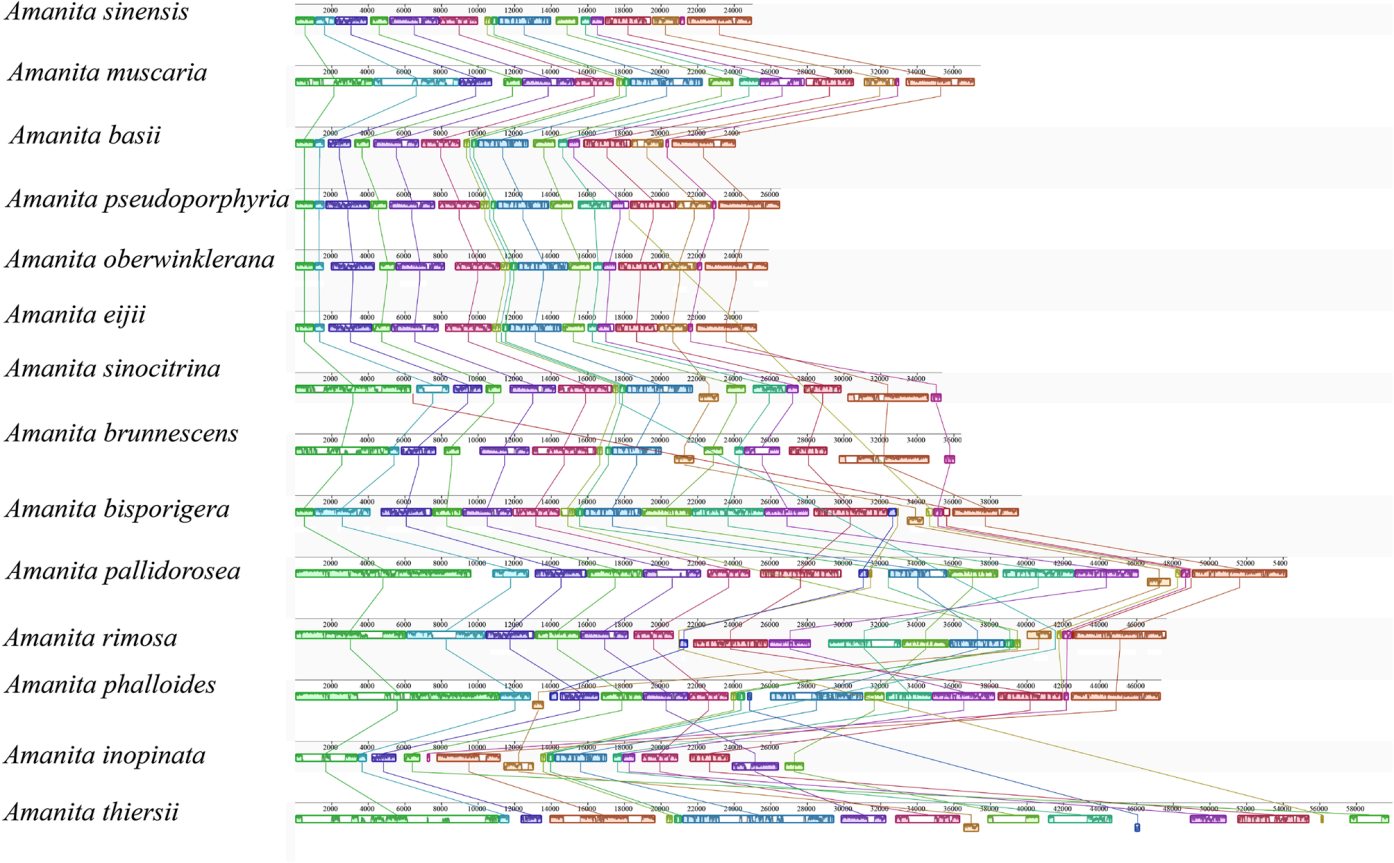
Genetic colinearity of 14 *Amanita* species on the basis of mitogenome sequence analysis using Mauve of Geneious R9

The mitogenomes of five *Amanita* were assembled, and the following statistics were obtained (Table 2): identical sites between 184 and 46,729 bp and pairwise identity between 98.2% and 99.7%. The mitogenomes of five *Amanita* species showed a double-stranded closed loop (Fig. 1). Most genes in the five *Amanita* species were located on the direct strand, with a small portion located on the reverse strand. The mitogenomes of 14 *Amanita* species (5 species of this study and 9 from NCBI) varied in size from 37,341 to 137,428 bp. Their AT and GC contents ranged from 73.8 to 76.9% and 23.1–27.6% respectively, and seven AT skews and two of the GC skews were negative (Fig. 2, Supplementary Material Tables S4, S5). The mitogenomes of the 14 *Amanita* species contained 15 PCGs, 26–27 tRNAs, and 2 rRNAs. Many tandem repeats were observed in each of the 14 *Amanita* species (Supplementary Material Table S6). The results showed good collinearity, but there were frequent irreversible structural reorganizations, which led to collinearity loss (Fig. 3). The genomic structure showed significant differences. There were many large sequence rearrangements and homologous regions, including 17 homologous regions and some local collinear regions. The size and arrangement of the homologous regions varied in different genomes, and a high degree of collinearity was observed in species with a closer phylogenetic relationships (e.g., *A*. *basii*, *A*. *eijii*, *A*. *muscaria*, *A*. *oberwinklerana*, *A*. *pseudoporphyria*, *A*. *sinensis* and *A*. *sinocitrina*).

**Table 2:** Raw NGS data and assembly statistics for five *Amanita* species

Feature	Species				
	Amanita eijii	Amanita oberwinklerana	Amanita pallidorosea	Amanita rimosa	Amanita sinocitrina
Total read bases	676,690,626	352,969,374	183,169,549	105,230,265	169,208,800
Total reads	4,510,920	2,352,748	1,220,465	700,896	1,127,575
GC (%)	26.1	24.5	27.2	25.2	32
Length (bp)	53,956	58,293	100,732	96,672	73,269

### Protein-coding gene analysis

Most of the PCGs in the five *Amanita* species were located on the direct strand and 15 PCGs of *A*. *pallidorosea* were located on the direct strand. A few genes of *A*. *eijii* (*cox3*), *A*. *oberwinklerana* (*cox3*), *A*. *rimosa* (*atp9*, *cob*, *cox3*, and *nad1*), and *A*. *sinocitrina* (*atp6*, *atp8*, *atp9*, *nad4L*, and *nad5*) were located on the reverse strand (Supplementary Material Table S3).

**Fig. 4:**
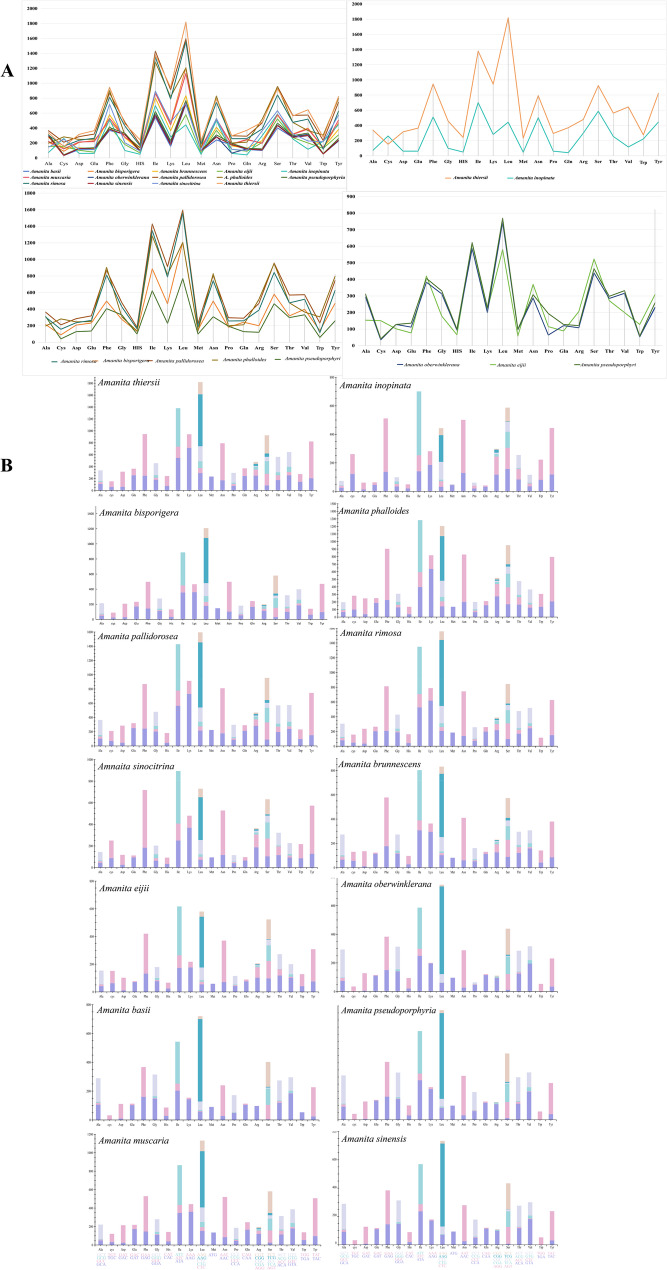
Amino acid distribution **A** and relative synonymous codon usage **B** of PCGs in 14 *Amanita* species. The data for *A*. *basii*, *A*. *bisporigera*, *A. brunnescens*, *A*. *inopinata*, *A*. *muscaria*, *A*. *pesudoporphyria*, *A*. *phalloides*, *A*. *sinensis*, and *A*. *thiersii* were obtained from the NCBI database

**Fig. 5:**
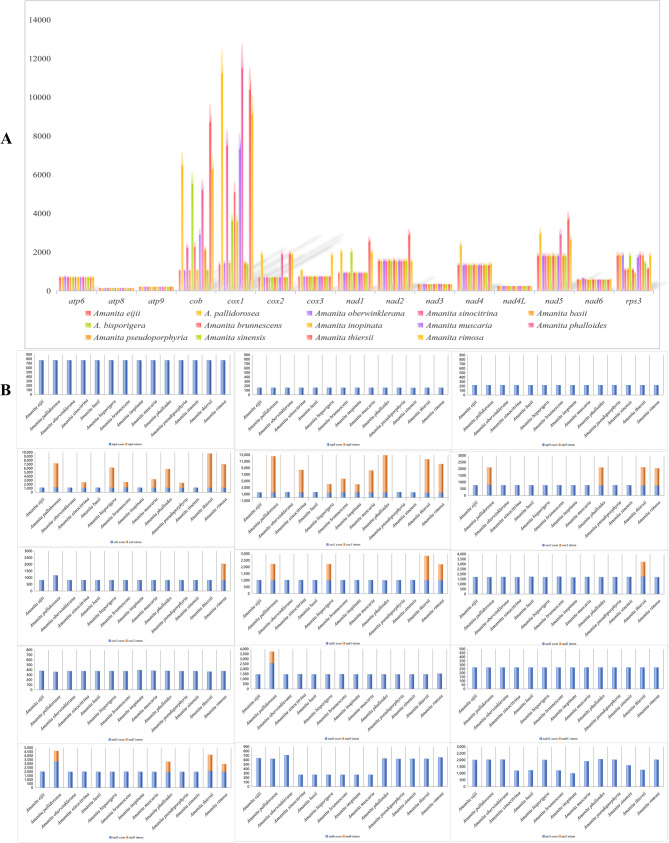
The length of 15 PCGs of 14 *Amanita* species. The data of *A*. *basii*, *A*. *bisporigera*, *A. brunnescens*, *A*. *inopinata*, *A*. *muscaria*, *A*. *pesudoporphyria*, *A*. *phalloides*, *A*. *sinensis*, and *A*. *thiersii* were obtained from the NCBI database

Most PCG start codons began with ATG and a few started with ATT, GTG, and ATA. The stop codons were TAA, TAG, and TGA. The frequently used amino acids were Leu, Ile, Ser, Phe, and Asn, and seldom used amino acids were Trp, Cys, His, Met, and Gln. Amino acid use was similar to the classification status, except for the two asymbiotic species (*A*. *inopinata* and *A*. *thiersii*). *Amanita pseudoporphyria* was closer to *A*. *oberwinklerana* of sect. *Roanokenses* (Fig. 4, Supplementary Material Table S5). The length of the 15 PCGs ranged from 159 bp (*atp8*) to 12,636 bp (*cox1*), and 23, 17, 8, 1, 8, 4, 1, 7, 20, and 22 introns were identified in *A. pallidorosea*, *A. rimosa*,* A*. *pseudoporphyria*, *A*. *bisporigera*, *A*. *brunnescens*, *A*. *inopinata*, *A*. *muscaria*, *A*. *phalloides*, *A*. *sinocitrina*, and *A*. *thiersii*, respectively; these species included *cox1*, *cox2*, *cob*, *nad1*, and *nad5* (Fig. 5, Supplementary Material Tables S3 and S7).

### rRNA and tRNA genes

**Fig. 6:**
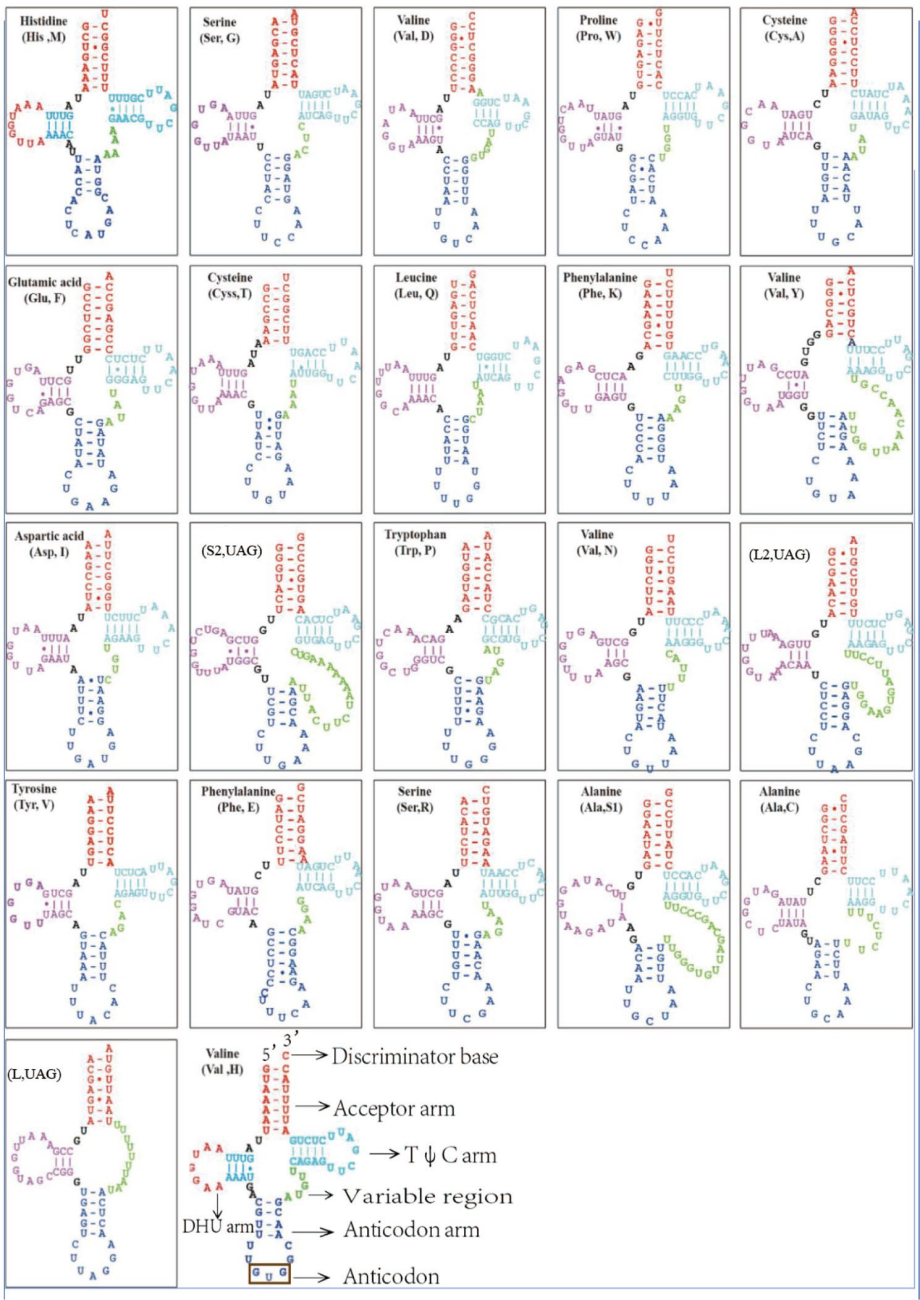
Second-level structure of tRNAs; the dot indicates GU nonclassical pairing

In the five *Amanita* species, 26–27 tRNA genes and 2 rRNA genes were identified. Two to three repeats were present for *trnR*, *trnI*, *trnM*, and *trnS*. Moreover, 27 tRNA genes were identified in *A*. *eijii* (3 *trnI*, 2 *trnL*, 3 *trnM*, 2 *trnQ*, 2 *trnR*, and 2 *trnS*), 27 in *A. pallidorosea* (3 *trnI*, 2 *trnL*, 3 *trnM*, 2 *trnR*, and 2 *trnS*), 27 in *A. oberwinklerana* (2 *trnL*, 3 *trnM*, 3 *trnR*, and 3 *trnS*), 27 in *A*. *sinocitrina* (3 *trnI*, 2 *trnL*, 3 *trnM*, 2 *trnR*, and 2 *trnS*), and 28 in *A. rimosa* (3 *trnI*, 2 *trnL*, 3 *trnM*, 2 *trnD*, and 2 *trnS*). *A*. *eijii* lacked *trnG* (Gly). We predicted the location and secondary structure using the MITOS Web Server (http://mitos.bioinf.uni-leipzig.de/index.py) and enhanced the image with Adobe Illustrator 2020 (Fig. 6). All tRNAs were 60–90 bp in length (*Supplementary Material Table S3*). Among the 26–27 tRNA genes identified from the five *Amanita* species, *trnL1* lacked the T Ψ C arm, which typically forms a loop. The remaining secondary structures were typical cloverleaf structures, with most anticodons corresponding to standard codons. We found one or two places of tRNA clusters that were closely associated with the relationship between species within the mitogenome. However, the degree of tRNA dispersion was positively correlated to rearrangements.

*rnl* genes were between 3,391 bp and 4,266 bp in length. *A*. *sinocitrina* was the longest (4,266 bp) and contained an intron, whereas *rns* ranged from 1,971 bp to 3,669 bp, with *A. pallidorosea* (3,669 bp) and *A. rimosa* (3,579 bp) containing one intron.

### Analysis of heterogeneity

**Fig. 7:**
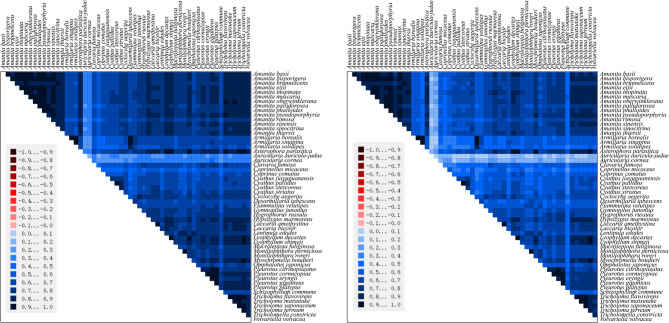
Heterogeneity of 15 PCGs in the mitogenome of 55 species of the Agaricomycetidae. Deep red to deep blue represents heterogeneity from heavy to Light. The left is the amino acids of 15 PCGs, and the right is the codons of 15 PCGs

Through pairwise comparison of the mitogenome dataset used for phylogeny and analysis of the base heterogeneity of the dataset, we preliminarily determined whether each data set could cause errors because of the heterogeneity of amino acids and codons composition in constructing the phylogenetic tree. Based on the results obtained using the Aligroove software, the overall heterogeneity in the mitogenome database was not high, with a higher base composition heterogeneity in the base dataset than in the amino acid dataset. The codon degeneracy contributed to reducing base compositional heterogeneity (Fig. 7).

### Phylogenetic analysis

A phylogenetic tree including 53 species of Agaricales and two species as an outgroup of Auriculariales was constructed using ML and BI methods Based on 15 PCGs (*Supplementary Material Table S2*). The results showed a nearly identical topology, with high support for most BI branches (BI posterior probabilities [BPP] > 1) and ML branches (bootstrap [BS] > 0.8). We conducted a comparative analysis of the mitogenome of Agaricales, which revealed the taxonomic status of *Amanita* within Agaricales. All species were grouped into 18 clades, corresponding to their taxonomic classification for the orders Amanitaceae, Pluteaceae, Tricholomataceae, Lyophyllaceae, Agaricaceae, Nidulariaceae, Hymenogastraceae, Bolbitiaceae, Hydnangiaceae, Psathyrellaceae, Schizophyllaceae, Marasmiaceae, Omphalotaceae, Physalacriaceae, Pleurotaceae, Hygrophoraceae, Clavariaceae, and Auriculariaceae. Fourteen *Amanita* species formed an independent clade attributed to sister branches with Pluteaceae. For the different families, *Amanita* showed the farthest genetic distance from Clavariaceae and Hygrophoraceae. *Pleurotus cornucopiae* and *P*. *platypus* in Pleurotaceae, and *Armillaria borealis* and *A*. *solidipes* in Physalacriaceae exhibited an equal genetic distance and belonged to the same species separately.

**Fig. 8:**
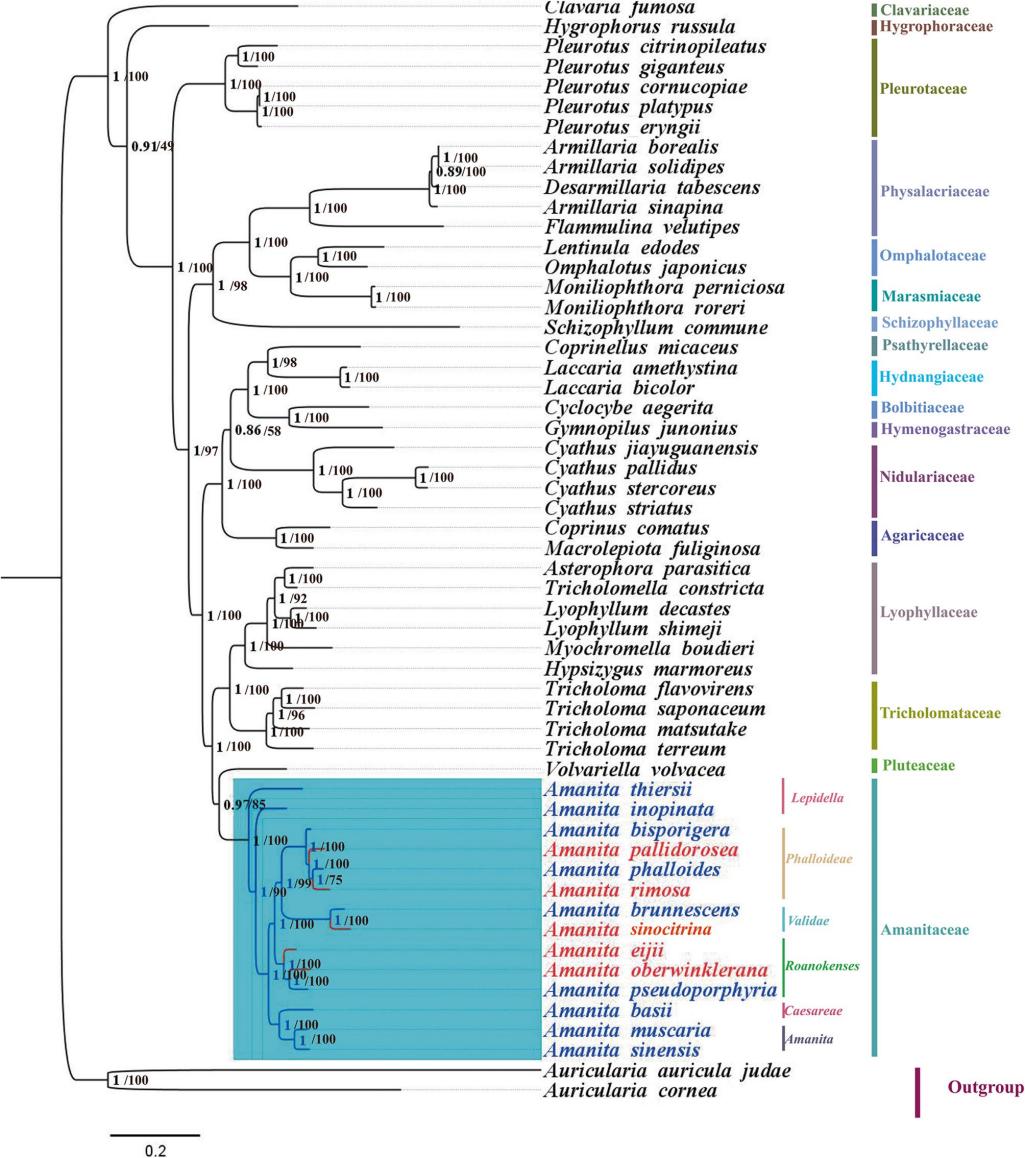
The maximum likelihood (ML) tree and Bayesian inference (BI) tree of Agaricales inferred from the 15 PCGs in the mitogenomes. ML bootstrap values over 40 and Bayesian posterior probabilities over 0.90 are shown above or beneath individual branches. The species labeled in red indicated the samples collected in this study

**Fig. 9:**
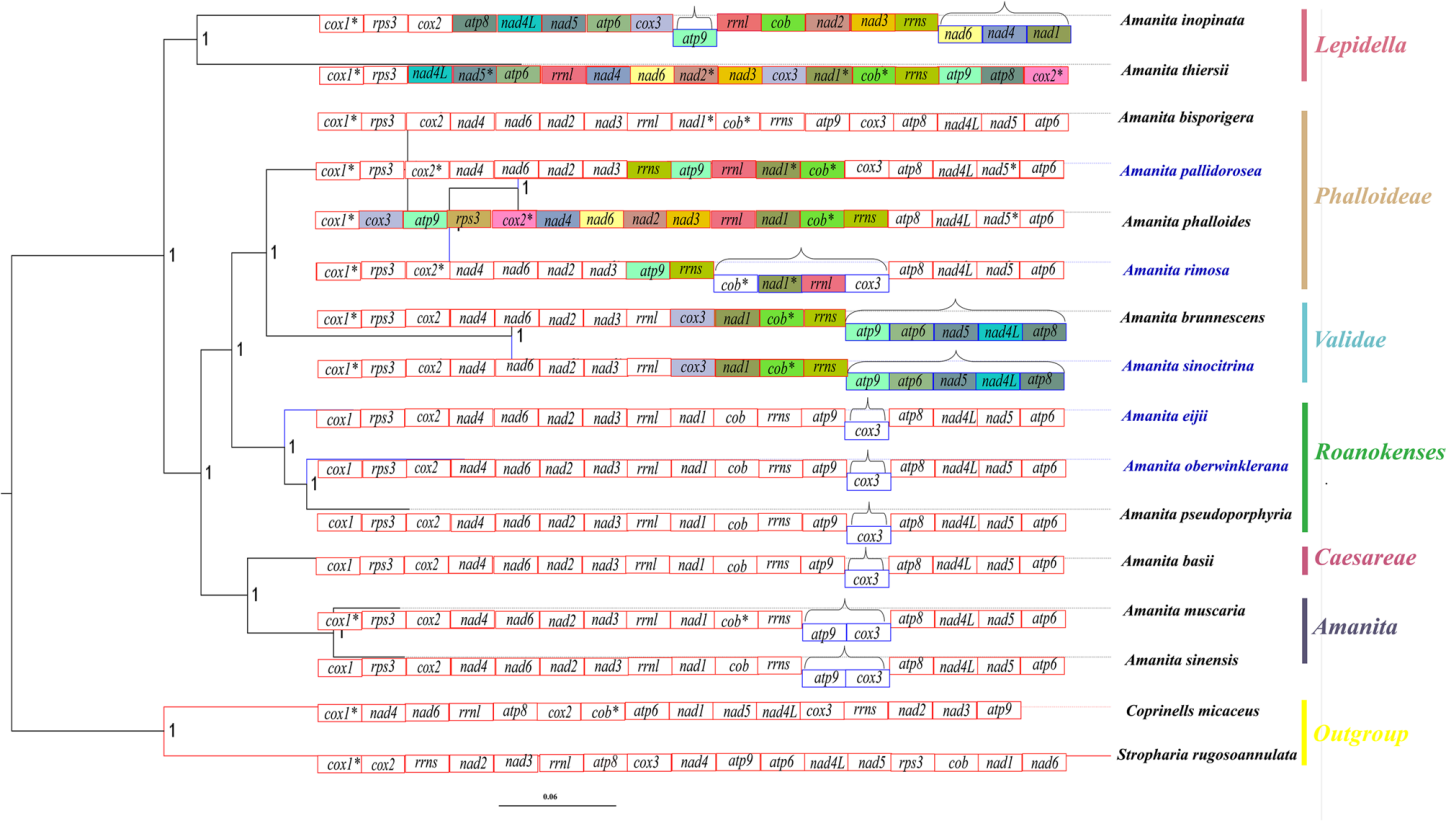
The phylogenetic tree of 14 *Amanita* species and 2 outgroup species (*Coprinellus micaceus* lacks the *rps3*) inferred from the Bayesian Inference analysis Based on the 15 PCGs in the mitogenomes, and the arrangement of 15 protein-coding genes and 2 rRNAs genes of 14 *Amanita* all starting from *cox1*. Different colors indicate different arrangements. Parentheses indicate the genes on the reverse strand and asterisks (*) represent the introns (indication does not include the rRNAs). Species names in blue indicate the samples collected in this study

**Fig. 10:**
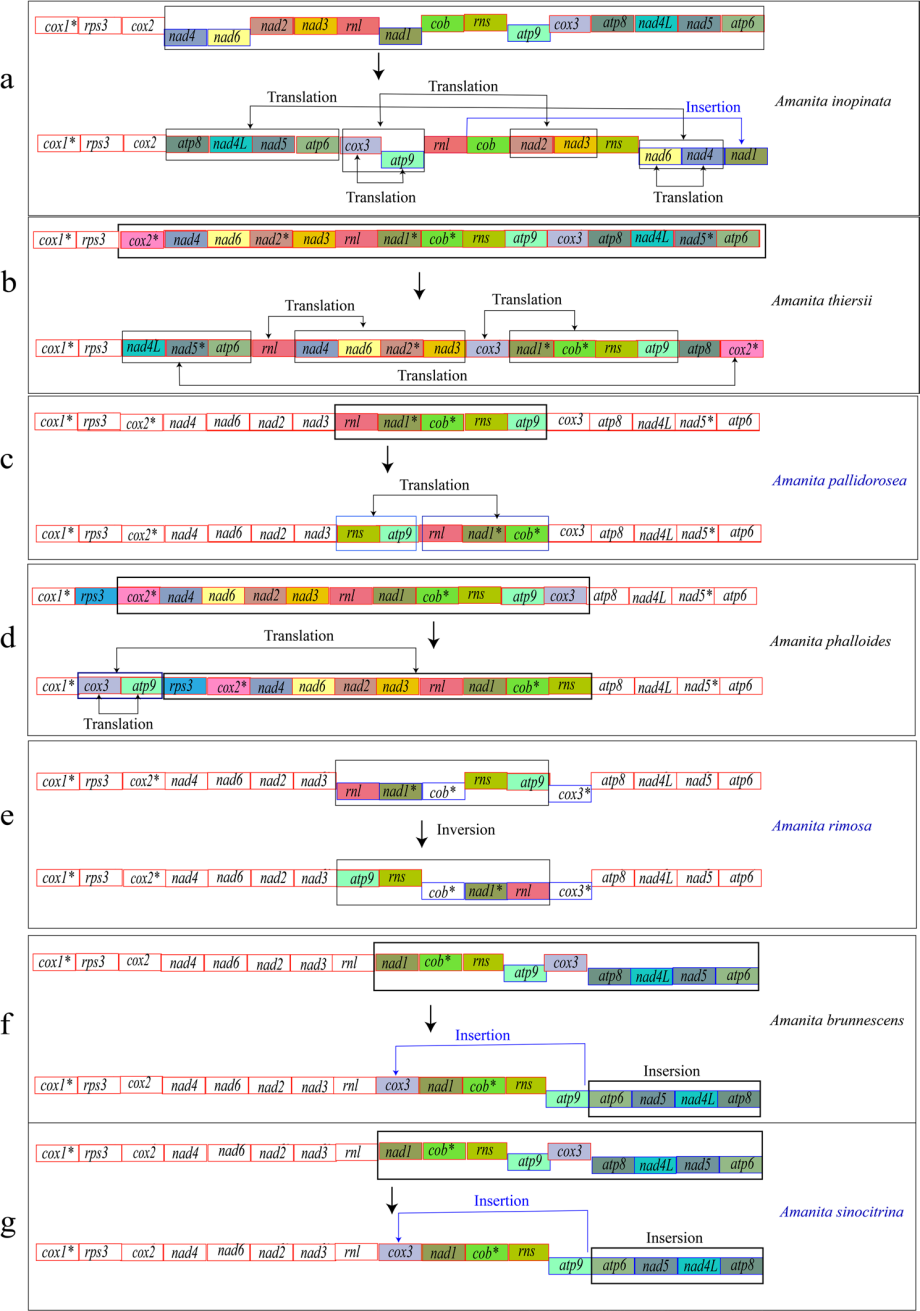
The rearrangements of 15 protein-coding genes and 2 rRNAs genes in the order of 7 *Amanita* species (*A. brunnescens*, *A*. *inopinata*, *A*. *pallidorosea*, *A*. *phalloides*, *A*. *rimosa*, *A*. *sinocitrina* and *A*. *thiersii*), all starting from *cox 1*. Different colors indicate different rearrangements, including translation, insertion, and inversion. The direction of the arrow indicates the position after gene change. Asterisks (*) in the genes represent the introns, and the species names in blue indicate the species from this study

The 14 *Amanita* species formed a single clade and were divided into two large subclades, which corresponded to asymbiotic and ectomycorrhizal organisms and aligned with two of the three subgenera of *Amanita* (Figs. 8 and 9). Among the 14 *Amanita* species, the phylogenetic positions of all species were identical to the current phylogeny. *Amanita pallidorosea*, *A*. *bisporigera*, *A*. *phalloides* and *A. rimosa* belonged to one section. *Amanita brunnescens* and *A*. *sinocitrina* were sister species. *Amanita eijii*, *A*. *oberwinklerana* and *A*. *pseudoporphyria* were clustered into one clade, and *A*. *oberwinklerana* and *A*. *pseudoporphyria* were sister species. *Amanita basii*, *A*. *muscaria*, and *A*. *sinensis* were closely related to each other, and *A*. *muscaria* and *A*. *sinensis* were sister species. Based on the order of gene arrangement: *A*. *eijii* and *A*. *oberwinklerana* were consistent with *A*. *basii*, *A*. *bisporigera*, *A*. *muscaria*, *A*. *pesudoporphyria*, and *A*. *sinensis* from NCBI; their genes were in the following order: *cox1*-*cox2*-*nad4*-*nad6*-*nad2*-*nad3*-*nad1*-*cob*-*atp9*-*cox3*-*atp8*-*nad4L*-*nad5-atp6*-*rps3*. The remaining species (*A. brunnescens*, *A*. *inopinata*, *A*. *pallidorosea*, *A*. *phalloides*, *A*. *rimosa*, *A*. *sinocitrina*, and *A*. *thiersii*) had one or more genes that were translated, inverted, or inserted at other locations. Gene rearrangement was correlated with phylogenetic position and genetic distance. The orders of the genes of the two mitogenomes of the asymbiotic *A*. *inopinata* and *A*. *thiersii* were completely disrupted and they had further evolutionary distances relative to the other sister clades in the phylogenetic trees of 14 *Amanita* species. *Amanita sinocitrina* and *A*. *brunnescens* (NC045197) published in GenBank belong to a section. At least three breakpoints existed in *A. brunnescens* and *A*. *sinocitrina*, occurring at *rnl* and *nad1*, *atp9* and *cox3*, and *cox3* and *atp8*. *cox3* was inserted between *rnl* and *nad1*. *atp8*, *nad4L*, *nad5* and *atp6* were inserted in an inverted position. At least nine breakpoints existed in *A*. *inopinata*, occurring between *cox2* and *nad4*, *nad6* and *nad2*, *nad3* and *rnl*, *rnl* and *nad1*, *nad1* and *cob*, *cob* and *rns*, *rns* and *atp9*, *atp9* and *cox3*, and *cox3* and *atp8*. *Nad4* and *nad6* switched position with parts of *atp8*, *nad4L*, *nad5*, and *atp6*. *Nad2* and *nad3*, as a whole, switched position with *atp9*, *cox3*, *atp9*, and *cox3*. *Nad1* was transferred from between *rnl* and *cob* to the end. *A*. *pallidorosea* had three breakpoints, which occurred between *nad 3* and *rnl*, *cob* and *rns*, and *atp9* and *cox3*. *rns* and *atp9*, as a whole, exchanged positions with *rnl*, *nad1*, and *cob*. *A*. *phalloides* had at least four breakpoints, which occurred between *cox1* and *rps3*, *rns* and *atp9*, *atp9* and *cox3*, and *cox3* and *atp8*. *Cox3* and *atp9* were inserted upstream of *rps3*, and the position of *cox3* was replaced with the position of *atp9*. At least two breakpoints exist in *A*. *rimosa*, which occur between *nad3* and *rnl* and *atp9* and *cox3*. *Rnl*, *nad1*, *cob*, *rns*, and *atp9*, as a whole, were inverted. At least five breakpoints existed in *A*. *thiersii*, which occurred between *cox2* and *nad4*, *nad3* and *rnl*, *rnl* and *nad1*, *atp9* and *cox3*, and *cox3* and *atp8*, with only position changes (i.e., no orientation changes) in these rearrangements (Figs. 9 and 10).

## Discussion

### Genomic structure and organization

The gene composition, Base composition, AT skews, and GC skews of 14 *Amanita* mitogenomes showed no significant differences or correlations with taxonomic status based on their amino acids and codons. The mitogenome size of *Amanita* showed great variation in Agaricales. The size of the 14 mitogenomes ranged from 37,341 to 137,428 bp. The factors affecting sequence length included genetic spacers, introns, and repetitive sequences [37–39].

The number of intergenic intervals in the mitogenome was higher in species with an asymbiotic phenotype than in ectomycorrhizal species [25]. Possibly, the asymbiotic is more influenced by the environment, and the genetic changes are more complex to cope with the changes in the environment and in order to avoid the interaction of diffrent genes, the intergenic intervals were correspondingly longer. The introns among the 15 PCGs were primarily found in *cob*, *cox1*, *cox2*, *nad1*, and *nad5*, which may have played a larger role in evolution. In the 14 *Amanita* species, only *A*. *rimosa* contains an intron in the *cox3* genes and the order of the genes also has a large variation. In other macrofungal mitogenomes, intron lengths exhibit a high degree of genetic diversity [39]. The diversity of introns is crucial in the evolution of the fungal mitogenome and facilitates evolutionary origin analysis. More introns can reduce the risk of avoiding the influence of adverse factors, such as mutations in coding genes, and the number of introns may be relatively higher in coding genes with important functions. The variation in genome size, intron number, and intergenic interval between the two asymbiotic species is very obvious, which may be a specific difference between asymbiotic and ectomycorrhizal species.

###  Tandem repeats and gene rearrangements

Numerous tandem repeats were identified in the mitogenomes of 14 *Amanita* species, ranging from a few bp to over 100 bp, with quantities ranging from several to dozens. There were more tandem repeats in the asymbiotic species [22], and the number was correlated to the size of the *Amanita* mitogenome. Most of the repeats were in genetic spacers, and these small repetitive sequences are likely characteristic of the species.

Many gene rearrangements, including translation, insertion, and inversion, were observed in the mitogenome of *Amanita*. This phenomenon is considered a morphological change in the genome in phylogenetic analysis. Mitogenome rearrangements are considered molecular data of evolution and are widely used in genomic studies of multiple biological taxa [40–42]. In the present study, many tandem repeats and gene rearrangements were identified in all *Amanita* species and the gene rearrangements were more pronounced in species with a high number of tandem repeats. The accumulation of repetitive sequences may lead to gene rearrangements and the rearrangement characteristics in related species exhibit high similarity [42]. However, the rearrangements differ greatly between the two asymbiotic species, possibly due to the large difference in evolution between the two species or the rearrangements among all asymbiotic species. In the mitogenomes of 14 *Amanita* species, all genes of *nad2*–*nad3* and *nad4L*–*nad5*, including genes in which rearrangements occurred, were always linked end to end and may be associated with last codon-sharing [43]. The degree of tRNA dispersion was positively correlated with rearrangements, and one or two tRNA clusters in the mitogenome were close. Conversely, the tRNA position may be altered and is related to the protein-coding order. Overall, tRNA distribution and dispersion distance are related to gene rearrangement [44]. In this study, we found that most tRNAs were distributed outside PCGs, and some tRNAs (*trnI*, *trnM*, *trnR*, and *trnS*) were in relatively large quantities; these codons were more frequently used in the genome. As large-scale gene rearrangements occurred, the mitogenome similarity between species gradually decreased. Thus, the application of mitogenome analysis proves to be advantageous in the study of phylogeny and evolution.

The mitogenome structure is complex because of massive rearrangement, which indicates its evolutionary importance [45, 46]. Collinearity analysis revealed a lower degree of collinearity and a higher rearrangement rate (Fig. 3). Angiosperm mitogenomes differentiate rapidly structurally, with frequent irreversible structural reorganization causing a loss of collinearity [46]. When a gene break reconnects, it results in gene loss or inactivation. In the present study, a high degree of collinearity was observed in species with closer taxonomic status and numerous rearrangements of homologous regions, which occurred in two asymbiotic species such as *A*. *inopinata* and *A*. *thiersii*, and resulted in the loss of collinearity. These two species belonged to one section and showed a close relationship; however, they exhibited great differences in many aspects. This could be due to a general difference in asymbiotic phenotypes or to the presence of smaller taxonomic units in this group. Given the limited data in this study, additional mitogenome data are needed to confirm whether gene arrangement order correlates with phylogenetic distance between related species. Further investigation is also warranted to determine whether gene rearrangement patterns differ between toxic and nontoxic *Amanita* species. This could offer new insights into the morphological differentiation of challenging species.

### Phylogenetic relationships

We reconstructed the phylogenetic tree of *Amanita* and Agaricales and observed the monophyly of *Amanita*. These results support the division of *Amanita* into ectomycorrhizal and asymbiotic species and its classification into three subgenera [1]. The 14 species were divided into 6 minor clades, which corresponded to sect. *Lepidella*, *Phalloideae*, *Validae*, *Roanokenses*, *Caesareae*, and *Amanita*. *Amanita inopinata* and *A*. *thiersii* of sect. *Lepidella* are asymbiotic [22, 46]. There were some differences in topologies between the two trees: in the phylogenetic tree of 55 species (Fig. 8), *A*. *inopinata* and *A*. *thiersii* were divided into two clades, whereas in the phylogenetic tree of 14 species (Fig. 9), the two species were clustered into one clade. We propose that the number of species probably affects the tree topology, and including more species may clearly resolve the phylogenetic relationships among species. Meanwhile, we know that there is a large gap between the two asymbiotic species relative to the ectomycorrhizal species in terms of genome size, intron number, repeat sequences, and rearrangement; however, with all species of sect. *Lepidella*, the sample volume was too small to confirm. We will require more data regarding the mitogenome to study the phylogenetic relationship, genetic taxonomy, and evolutionary analysis of *Amanita* and other related species.

The number of introns is correlated with the evolutionary distance and influences the evolution of a species to a certain extent. The original ancestors of the introns remain unknown, and some species lack introns. In plants, intron-less species genes are younger than intron-containing genes, which are derived from intron-less genes [47]. We believe that the mitogenome is inherited differently from the nuclear genome and that some of the mutations that occur during the inheritance of the mitogenome do not accumulate in the nuclear genome [48]. A high number of tandem repeats and gene rearrangements in asymbiotic species explain this phenomenon, although more mitogenome data are needed to confirm this. The gene order in the mitogenome can reflect phylogenetic information to some extent. The rearrangement of PCGs is related to the classification status, showing a different arrangement between asymbiotic sections and the rearrangement of PCGs with introns is more apparent [42, 49]. The asymbiotic species showed a clear difference from other species of *Amanita*. In the asymbiotic *Amanita* and ectomycorrhizal *Amanita*, there is no correlation between nuclear genes and gene size, gene rearrangement, repetitive sequences, or transposon content. The transition from an asymbiotic *Amanita* to ectomycorrhizal *Amanita* does not require the production of a lot of new genes, nor is it due to gene loss; rather, it may be caused by different ecological environments and gene arrangements [50]. In this study, there was no significant difference between the size of the two asymbiotic *Amanita* mitogenomes with the ectomycorrhizal species mitogenome.

We believe that there are still controversial problems in the subordinate taxon of *Amanita*, such as the different taxonomic statuses explained above. These questions will be addressed as additional mitogenome data are accumulated. Mitogenomes as a comparative advantage of molecular data, can be used to solve the limitations of the small molecular fragments. Because of the long-term influence of various factors, such as environmental conditions, organisms, and autotoxins, toxic and edible species exhibit different gene rearrangement patterns and phylogenetic relationships, resulting in distinct distributions. This can provide a new method for distinguishing between toxic and edible *Amanita* species. By integrating information from multiple PCGs, mitogenome data provided a more powerful and detailed evolutionary framework and a more comprehensive and supported of evolutionary relationships.

## Conclusion

*Amanita* species exhibit considerable variation in both the length of their mitogenomes and the structure and organization of protein-coding genes. For the taxonomic identification of difficult *Amanita* species, compared with the traditional morphological classification, molecular biology technology can produce taxonomic evidence at the molecular level. The presence of multiple variants and other complex situations imposes challenges while comparing the highly similar sequences of small molecule fragments with the complexity of the complete genome. The mitogenome contains several functional genes, and the evolution rate is fast. Moreover, as an outer nuclear genome, the mitogenome has several advantages in species classification and evolutionary analysis. In this study, the phylogenetic relationships of 14 mitogenomes were similar to those of the previously described taxonomic status. To obtain clearer taxonomic relationships, more mitogenome data are needed. Mitogenome data for the *Amanita* genus are limited, even for the whole Basidiomycota, to reveal the evolutionary and phylogenetic relationships among fungi. Additional mitogenome data will provide strong evidence for more species with an unclear and controversial classification status, thus offering further insights into the diversity of macrofungi at the genetic level.

## Supplementary Information

Supplementary Material 1.

## Data Availability

The mitogenomes of five Amanita species (Amanita eijii, Amanita oberwinklerana, Amanita pallidorosea, Amaita rimosa, and Amanita sinocitrina) have been deposited in GenBank under the accession numbers PP409005, PP409003, PP978973, PP978972, and PP409004, respectively. Other mitochondrial genomes were downloaded from GenBank, and their accession numbers are listed in Supplementary Material Table S1. The raw NGS reads data have been deposited in GenBank under the bioSample accessions: SAMN46046466, SAMN46046467, SAMN46046468, SAMN46046469, and SAMN46046470. Other supporting results are included within the article and its additional files.

## Abbreviations

Mitogenome: Mitochondrial genome
AT: Adenine-thymine
GC: Guanine-cytosine
tRNA: Transfer RNA
bp: Base pair
cob: Cytochrome b
PCG: Protein coding gene
rRNA: Ribosomal RNA
tRNA: Transfer RNA
rnl: large subunit ribosomal RNA
rns: small subunit ribosomal RNA
NGS: Next generation sequencing
atp6: ATPase subunit 6
atp8: ATPase subunit 8
Atp9: ATPase subunit 9
cox1–3: Cytochrome oxidase subunit 1–3
nad1–6: NADH dehydrogenase subunit 1–6
nad4L: NADH dehydrogenase subunit 4 L
ML: Maximum likelihood
BI: Bayesian inference
gDNA: Genome DNA
rDNA-ITS: Ribosomal DNA Internal Transcribed Spacers
ITS1: Internal Transcribed Spacers 1
